# Association between Bacterial Vaginosis and Cervical Intraepithelial Neoplasia: Systematic Review and Meta-Analysis

**DOI:** 10.1371/journal.pone.0045201

**Published:** 2012-10-02

**Authors:** Evy Gillet, Joris F. A. Meys, Hans Verstraelen, Rita Verhelst, Philippe De Sutter, Marleen Temmerman, Davy Vanden Broeck

**Affiliations:** 1 International Centre for Reproductive Health (ICRH), Ghent University, Ghent, Belgium; 2 Department of Gynaecology, Vrije Universiteit Brussel, Brussels, Belgium; 3 Department of Applied Mathematics, Biometrics and Process Control, Faculty of Bioscience Engineering, Ghent University, Ghent, Belgium; 4 Department of Obstetrics and Gynaecology, Ghent University Hospital, Ghent, Belgium; University of Buea, Cameroon

## Abstract

**Objective:**

Bacterial vaginosis (BV), the most common vaginal disorder among women of reproductive age, has been suggested as co-factor in the development of cervical cancer. Previous studies examining the relationship between BV and cervical intra-epithelial neoplasia (CIN) provided inconsistent and conflicting results. The aim of this study is to clarify the association between these two conditions.

**Methods:**

A systematic review and meta-analysis were conducted to summarize published literature on the association between BV and cervical pre-cancerous lesions. An extensive search of electronic databases Medline (Pubmed) and Web of Science was performed. The key words ‘bacterial vaginosis’ and ‘bacterial infections and vaginitis’ were used in combination with ‘cervical intraepithelial neoplasia’, ‘squamous intraepithelial lesions’, ‘cervical lesions’, ‘cervical dysplasia’, and ‘cervical screening’. Eligible studies required a clear description of diagnostic methods used for detecting both BV and cervical pre-cancerous lesions. Publications were included if they either reported odds ratios (OR) and corresponding 95% confidence intervals (CI) representing the magnitude of association between these two conditions, or presented data that allowed calculation of the OR.

**Results:**

Out of 329 articles, 17 cross-sectional and 2 incidence studies were selected. In addition, two studies conducted in The Netherlands, using the national KOPAC system, were retained. After testing for heterogeneity and publication bias, meta-analysis and meta-regression were performed, using a random effects model. Although heterogeneity among studies was high (χ^2^ = 164.7, p<0.01, I^2^ = 88.5), a positive association between BV and cervical pre-cancerous lesions was found, with an overall estimated odds ratio of 1.51 (95% CI, 1.24–1.83). Meta-regression analysis could not detect a significant difference between studies based on BV diagnosis, CIN diagnosis or study population.

**Conclusions:**

Although most studies were cross-sectional and heterogeneity was high, this meta-analysis confirms a connection between BV and CIN.

## Introduction

Cervical cancer, the second most common malignity after breast cancer among women worldwide, is responsible for more than half a million new cases and a quarter of a million deaths annually [1]. Despite impressive progress in prevention strategies, the burden of this disease remains a significant health problem, especially in developing countries.

Research has established the causal role of oncogenic human papillomavirus (HPV) infection in the pathogenesis of invasive cervical cancer and its precursor lesions, i.e. cervical intraepithelial neoplasia (CIN) [2]. However, HPV infection is widely prevalent among sexually active women and mostly self-limiting, causing no or only mild and transient cytological abnormalities. Just a small proportion of HPV-infected women will eventually develop cervical cancer, suggesting involvement of additional host or external factors acting together with HPV in cervical carcinogenesis [3].

Identifying risk factors for the development of CIN and cervical cancer has been the objective of several studies. Progression to precancerous cervical lesions by HPV seems to depend on the infecting virus genotype (HPV types 16 and 18 cause approximately 70% of all cervical cancers worldwide [4]) and co-infection with multiple HPV-types. Persistent HPV infection is a prerequisite for progression to high-grade lesions [4] and HPV infection can result in malignancy if the immune system is not able to clear this virus [5]. In addition, epidemiologic investigation shows that there are numerous risk factors for CIN and cervical cancer, such as young age at first intercourse, multiple sexual partners, cigarette smoking, race, high parity, oral contraceptive use, and low socioeconomic status [6]–[8]. Infections with sexually transmitted agents, such as Chlamydia trachomatis, herpes simplex virus, and human immunodeficiency virus (HIV), have been proposed as co-factors likely to influence the risk of progression from cervical HPV infection to high-grade lesions and cervical cancer [8], [9].

It has been suggested that bacterial vaginosis (BV), the most common vaginal disorder among women of reproductive age, may play a role in cervical carcinogenesis. It has been noted that cervical cytological abnormalities are found significantly more often in women with a disturbed vaginal flora, suggesting a possible link between BV and the development of cervical cancer [10]–[14]. BV is characterized by a shift from the protective *Lactobacillus*-predominant vaginal flora to an overgrowth of anaerobic bacteria, including *Gardnerella vaginalis, Atopobium vaginae, Mobiluncus* species, and *Prevotella* species. This disturbance in the vaginal microenvironment leads in about half of the cases to the clinical presentation of a malodorous discharge, an elevated vaginal pH, a positive amine ‘whiff’ test and the presence of clue cells on a wet smear [15]. Although the cause of BV is unknown, predisposing factors include sexual intercourse, cigarette smoking, vaginal douching, use of uterine devices and black ethnicity [16]. This infestation is known to be associated with many gynaecologic and obstetric complications, such as pelvic inflammatory disease (PID), postoperative infections, cervicitis, preterm labour and delivery, chorioamnionitis, and premature rupture of membranes [17].

Evidence regarding an association between BV and cervical pre-cancerous lesions has so far been conflicting and is still a matter of debate. Results of previous studies examining the relationship between BV and CIN ranged from a very strong association between the two conditions, as described in a retrospective study by Platz-Christensen et al [13] (relative risk of 5.0 for CIN III in women with BV; 95% CI 2.2 to 11.6), to no association at all as in the study by Peters et al [18].

The goal of this meta-analysis is to systematically review all published studies on the association between BV and cervical pre-cancerous lesions, and to analyze the eligible data to assess an estimate of association between these two conditions. This meta-analysis takes into account most prominent sources of heterogeneity regarding the relationship between BV and CIN.

## Methods

### Literature search

Relevant studies on the association between BV and cervical pre-cancerous lesions were identified through an extensive search of the electronic databases Medline (Pubmed) and Web of Science, based on following key words: ‘bacterial vaginosis’, ‘bacterial infections and vaginitis’ in combination with ‘cervical intraepithelial neoplasia’ (CIN), ‘squamous intraepithelial lesions’ (SIL), ‘cervical lesions’, ‘cervical dysplasia’, and ‘cervical screening’. In addition, reference lists of retrieved papers and reviews were further examined to identify any articles missed by this initial search. Studies that examined the relationship between BV and CIN or SIL were reviewed through predefined eligibility criteria.

Included studies needed a clear description of diagnostic methods used for detecting both BV and cervical pre-cancerous lesions. Articles were selected if they either reported odds ratios and corresponding 95% confidence intervals (CI) representing the magnitude of association between BV and cervical pre-cancerous lesions or presented data that allowed calculation of the OR. Initial search had no limitations on study design.

Literature search stopped in December 2009, but there was no publication starting-date limitation. The meta-analysis was restricted to original articles (no expert opinions, editorials or reviews). Conference abstracts and other unpublished articles were also excluded. Studies were restricted to those written in English. Two authors (EG and DVB) verified inclusion criteria independently and reached consensus in case of discordance. Reporting of this meta-analysis was based on the PRISMA Guidelines (Preferred Reporting Items for Systematic reviews and Meta-Analysis) [19]. Raw data are provided under Annex 1.

### Data abstraction and selection criteria

For each study, following data were extracted: year of publication, first author, country and year(s) during which the study was conducted, number of cases enrolled, study population, age range of participants, method of CIN diagnosis, grade of CIN lesions, BV diagnostic criteria and BV prevalence.

Study populations were categorized in 2 groups: women screened for cervical cancer or premalignant lesions, and women with an indication smear. The latter included women referred to a colposcopy clinic because of previous abnormal Pap-smear, women attending obstetrics/gynaecology clinics or mixed patient groups (referred women, attendees and/or screened women). Two studies following HIV positive women were also categorized in the indication group [20], [21], since women with HIV are at higher risk for cervical intraepithelial lesions.

Diagnostic criteria for BV included Nugent's scoring system (BV when score ≥7), Amsel clinical criteria, modified Amsel criteria and presence of clue cells [15], [22], [23]. In the most accurate method of Nugent's scoring system, Gram-stained vaginal smears are assessed for average number of bacterial morphotypes seen per oil immersion field with large gram-positive rods (*Lactobacilli*) being scored inversely from 0 to 4, small gram-variable or gram-negative rods (*Gardnerella* and *Bacteroides* spp) from 0 to 4 and curved gram-variable rods (typically *Mobiluncus* spp) scored from 0 to 2 [15]. Amsel criteria define BV as presence of at least any three of following characteristics: homogeneous white-grey discharge that sticks to the vaginal walls; vaginal fluid pH >4.5; release of fishy amine odour from vaginal fluid when mixed with 10% potassium hydroxide (positive whiff test); and clue cells visible on wet mount microscopy [23]. Modification of Amsel criteria confirmed BV when only two of these four elements were present [22]. Studies detecting BV only through presence of clue cells on wet smear or more than 20% clue cells on Papanicolaou smear were also included, since this is confirmed by previous studies to be an accurate method [24].

Studies eligible for inclusion defined cervical precancerous lesions according to the CIN histology system (CIN I–III), or the Bethesda cytology system (low-grade SIL and high-grade SIL; ASCUS or Atypical Squamous Cells of Undetermined was not included).

Odds ratios giving an association between BV and cervical dysplasia and their respective standard errors were also retrieved from studies conducted in the Netherlands using the Dutch national coding system for cervical cytology (KOPAC). This system provides the opportunity to study the status of the squamous epithelium (P1–P9, with P4  =  LSIL and >P5  =  HSIL) concurring with inflammatory events (O3  =  dysbacteriosis, defined as detection of clue cells) [25]–[27].

Table 1 and 2 (the latter including studies using the Dutch KOPAC system) describe the characteristics of included studies ranked by year of publication.

**Table 1:** Overview of studies included in meta-analysis BV – CIN.

Year of publication	Authors	Country	Study year(s)	Nr cases enrolled	Participants	Age range (in years)	Cervical lesion diagnosis	Grade cervical lesions	BV diagnosis	BV prevalence
1985	Guijon et al [42]	Canada	-	87	mix	-	Hist	CIN I–III	Amsel	34.94%
1992	Guijon et al [11]	Canada	-	185	mix	17–33	Hist	CIN I–III	Amsel	36.76%
1993	Kharsany et al [12]	South Africa	-	48	referred	18–52	Hist	CIN I–III	Amsel	37.50%
1994	Platz-Christensen et al [13]	Sweden	Archival smears from 1976	6150	screened	32–36	Cyt	CIN I–III	Clue cells	10.03%
1995	Eltabbakh et al [40]	US	Jan 1991–Jan 1994	963	attendees	13–78	Cyt	LSIL–HSIL	Amsel	28.87%
1995	Peters et al [18]	The Netherlands	Sep 1988–Sep 1993	280	referred	20–66	Hist	CIN I–III	Mod Amsel	20.00%
1997	Barrington et al [10]	UK	-	200	mix	17–55	Hist	CIN I–III	Clue cells	22.50%
1997	Frega et al [41]	Italy	-	1.008	attendees	17–60	Hist	CIN I–III	Amsel	42.46%
1998	Uthayakumar [44]	UK	Jan 1991 and July 1994	300	attendees	-	Hist	CIN I–III	Amsel	24.49%
2000	Schiff et al [14]	US	Nov 1994–Oct 1997	628	mix	18–45	Hist	CIN II–III	Nugent	36.84%
2001	Castle et al [36]	Costa Rica	1993–1994	8.582	screened	-	Hist	High-grade lesions	Nugent	34.51%
2002	Behbakht et al [37]	US	Jan 2000–Oct 2000	51	mix	13–65	Hist	Dysplasia	Nugent	49.02%
2003	Boyle et al [38]	UK	Sept 1996–June 1998	379	attendees	16–58	Cyt	Dyskaryotic smears	Amsel	32.45%
2006	Discacciati et al [39]	Brazil	-	220	attendees	-	Cyt	LSIL-HSIL	Clue cells	15.00%
2006	Spinillo et al [21]	Italy	Jan 1996–Dec 2004	216	HIV attendees	-	Hist	CIN I–III	Amsel	17.27%
2007	Vetrano et al [45]	Italy	1991–2003	504	attendees	18–61	Hist	CIN I–III	Amsel	41.67%
2008	Lehtovirta et al [20] **	Finland	Jan 1989–May 2006	153	HIV attendees	-	Hist	CIN I–III	Clue cells	24.18%
2009	Nam et al [43]	South-Korea	Sep 2002–May 2006	510	mix	-	Hist	CIN I–III	Amsel	10.98%

** Incidence study.

Abbreviations: CIN =  Cervical Intraepithelial Neoplasia, Hist =  Histology, Cyt =  Cytology, LSIL =  Low-grade Squamous Intraepithelial Lesion, HSIL =  High-grade Intraepithelial Lesion, BV =  Bacterial Vaginosis, Mod Amsel =  Modified Amsel criteria.

Participants: referred (women referred to colposcopy clinic because of abnormal Pap-smear), attendees (women attending family planning or obstetrics and gynaecology clinics), screened (population sample, screening), mix (referred, attendees and/or screened); two studies include HIV-infected women.

**Table 2:** Overview of Dutch studies using the KOPAC system.

Year of publication	Authors	Study year(s)	Nr smears or ♀ enrolled	Indication smear	Age range (in years)	Diagnosis CL	Grade CL	BV diagnosis	BV prevalence
2009	Roeterset al [26]	1991–2008	1.008.879 smears	screening	18–72	Cyt (P5–P9)	HSIL	dysbacteriosis --> ‘clue cells’	3.14%
2009	Roeterset al [26]	1991–2008	1.008.879 smears	Symptoms or follow-up	18–72	Cyt (P5–P9)	HSIL	dysbacteriosis --> ‘clue cells’	5.48%
2007	Engbertset al [25] **	1991–2003	100.605 ♀	screening	30–60	Cyt (P4–P9)	LSIL-HSIL	dysbacteriosis --> ‘clue cells’	5.27%
2006	Verbruggen et al [27]	1995–2002	445.080 smears	screening	30–60	Cyt (P4–P9)	LSIL-HSIL	dysbacteriosis --> ‘clue cells’	3.61%

** Incidence study.

Abbreviations: CL =  Cervical Lesions, Cyt =  Cytology, P =  Plaveiselcelepitheel (KOPAC system), LSIL =  Low-grade Squamous Intraepithelial Lesion, HSIL =  High-grade Intraepithelial Lesion, BV =  Bacterial Vaginosis.

### Statistical analysis

Meta-analysis was conducted for studies fulfilling above-reported criteria, using packages for STATA provided by Sterne and colleagues [28]. The summary estimate was based on calculation of odds ratios for 18 cross-sectional studies. For one study the crude odds ratios as reported in the article were used [26]. Because most included studies had a cross-sectional design, only odds ratios could be used, as they do not assume a causal relation in one direction. Two incidence studies were not included in the meta-analysis [20], [25].

Possible publication bias was examined graphically, using funnel plots. The asymmetry of funnel plots was statistically evaluated using the Begg rank correlation test [29]. Heterogeneity between studies was assessed using Cochran Q test [30] and further quantified by the statistic I^2^ according to Higgins and Thompsons [31], defined as the percentage of total variation across studies attributable to heterogeneity.

Due to the presence of a significant degree of heterogeneity, the random effects model of DerSimonian and Laird was preferred for pooling odds ratios and determining the estimate of association between BV and CIN [32]. Results were visualised in a forest plot. The impact of each study on the summary estimate was explored using influence analysis, in which the meta-analysis estimates are computed omitting one study at a time and obtaining a summary for all the other studies.

To investigate possible sources of heterogeneity and their effect on the overall OR estimate, meta-regression was performed using the restricted maximum likelihood framework. The calculations were performed using the metafor package in R [33], implemented by Viechtbauer [34]. The same approach was used to calculate the pooled BV prevalence based on the reported raw data. Pooled BV prevalence was calculated with a random effect model using restricted maximum likelihood via the metafor package. Effects of diagnostic criteria and study population on the BV prevalence was estimated with a mixed effect model, again using restricted maximum likelihood. Given the heterogeneity, the standard error on the estimate was adjusted using the method of Knapp and Hartung [35]. In all analyses, the raw frequencies were used as input for the mentioned methods, both using Stata and using R.

## Results

### Study Inclusion Criteria and Characteristics

**Figure 1:**
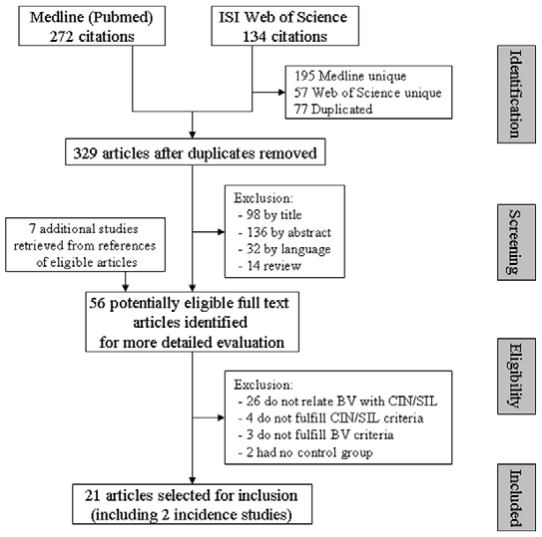
Study selection flowchart for meta-analysis BV – CIN.

Initial search of databases Medline and Web of Science yielded respectively 272 and 134 publications, a total of 329 unduplicated articles. Titles and abstracts from these publications were reviewed. Fifty-six articles were considered of interest and retained for more detailed evaluation, of which 21 were finally retrieved for further analysis. One study (Roeters et al, 2009) was used twice, once to extract screening data and once to derive follow-up data. Figure 1 summarizes the study selection process.

Most studies did not focus specifically on the association between BV and cervical lesions, and BV was often just an additional condition evaluated during gynaecological visits. The majority of studies did not use adjusted odds ratios (AOR) or did not describe clearly potential confounders. Therefore, only raw frequencies were retrieved and odds ratios (OR) with 95% confidence intervals (CI) were calculated, without adjustment for confounding factors. Three studies performed multivariate analysis: Castle et al [36] (N = 142) found an AOR of 0.84 with 95% CI 0.37–1.6 (adjusted for age, number of pregnancies and number of cigarettes) for the association between BV and cervical lesions, Schiff et al [14] (N = 437) an AOR of 1.6 with 95% CI 1.0–2.7 (adjusted for age, age at first intercourse, lifetime number of sex partners), and Spinillo et al [21] (N = 566) an AOR of 1.55 with 95% CI 1.0–2.39 (adjusted for CD4 <200/mm^3^ and detectable blood HIV-1 RNA). Considering the variation and limited adjustment for confounding factors, meta-analysis was performed with raw data. One original paper did not provide these raw data, hence crude odds ratios and the reported standard error were used [26].

All studies included in the meta-analysis were prevalence studies. Most of these studies were cross-sectional, assessing BV and CIN prevalence at a given point of time [10]–[14], [18], [36]–[45]. Two studies conducted in the Netherlands, using the KOPAC system for screening, had a longitudinal design, assessing prevalence data [26], [27]. Two incidence studies were found, defined as recruiting CIN-negative women and prospectively measuring incidence of CIN in women with and without BV. Both studies showed an increased risk of CIN/SIL in BV positive women (*Lehtovirta et* *al* Hazard ratio 1.85, 95% CI 1.04–3.28; *Engberts et* *al* OR 1.89, 95% CI 1.42–2.52) [20], [25].

Nineteen prevalence studies fulfilling the eligibility criteria and providing data on the association between BV and CIN/SIL, were included for meta-analysis, representing a total of 11.556 women, and an additional 1.453.959 smears analyzed by the Dutch KOPAC system. These studies reported 25 different estimates of association between BV and CIN/SIL prevalence for nineteen study populations (four studies reported estimates using two different BV diagnostic criteria, one study reported estimates using two different methods to diagnose cervical lesions). The estimate based on the most accurate method was used for meta-analysis. For BV diagnosis Nugent's score was preferred above Amsel [37] and presence of clue cells [14], and Amsel above Schröder (Grade III) criteria [11], [42]. For diagnosing cervical lesions, histology was preferred above cytology [44]. However, a clear description of the grade of cervical lesions was not always the case. Most studies described CIN grade I–III, or LSIL and HSIL (LSIL can be compared with CIN grade I, HSIL with CIN grade II and III). Two studies focused only on high-grade lesions [26], [36]. One study separately studied the association between BV and CIN II–III (raw data mentioned), and between BV and CIN I [14]. From the latter, no raw data were described, only an odds ratio adjusted for age, age at first intercourse and lifetime number of sex partners was mentioned in the article (AOR 2.0; 95% CI, 1.3–2.9). One study was included twice, because the association between BV and cervical lesions was evaluated separately on screening and indication smears (symptomatic or referred women) [26]. It cannot be ruled out that some women were included in both studies, given the fact that data was collected over a period of 18 years.

Regarding geographical location, ten studies were conducted in Europe, five in North-America (USA or Canada), one in Latin-America, one in Asia, and one in Africa. Four studies were conducted in developing countries (including 920 women), the others in developed countries (including 10.636 women and 1.453.959 smears analysed by the KOPAC system).

### Diagnosis and Prevalence of Bacterial Vaginosis

Studies included in the meta-analysis diagnosed BV using clinical Amsel criteria in 10 out of 19 studies [11], [12], [21], [38], [40]–[45], Nugent's score in 3 out of 19 studies [14], [36], [37], and presence of clue cells in 5 out of 19 studies (including two studies using the KOPAC system) [10], [13], [26], [27], [39]. One study used modified Amsel criteria, diagnosing BV by the combination of presence of clue cells and a positive amine whiff test [18].

BV prevalence ranged from 3.14% in asymptomatic women aged between 18 and 72 years screened in The Netherlands (BV diagnosed using the national KOPAC system) [26] to 49% in women aged 13 to 65 years referred to colposcopy clinic and OB/Gyn attendees in the USA (BV diagnosed by Nugent criteria) [37]. Large variation in reported prevalence figures may be due to inclusion of different patient populations, demographical variation, and variation in diagnostic criteria. The pooled BV prevalence was 27.1% (95% CI, 20.7%–33.4%). Heterogeneity in BV prevalence among the studies was substantial according to Cochran's Q test (χ^2^ = 2292; p<0.01). More than 99% of the observed variance can be explained by heterogeneity (I^2^ = 99.43%). The study of *Roeters et* *al.* was excluded to calculate this prevalence, as the lack of raw data did not allow to calculate the standard error on their prevalence estimate.

**Figure 2:**
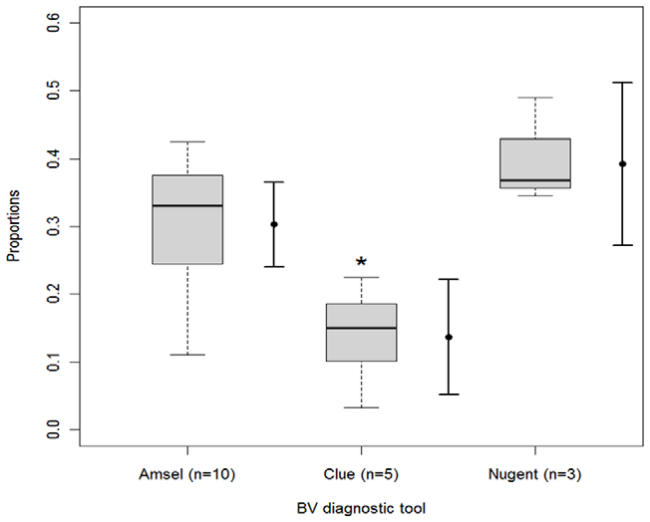
Box plots showing the difference in BV prevalence depending on BV diagnostic criteria (Amsel, Clue cells only and Nugent). Comparison between data distribution (box plots) and estimated average (full vertical line).

Differences in BV prevalence were significant according to diagnostic criteria used (p = 0.0003). BV prevalence did not differ significantly between studies using Nugent's criteria (39.2%, 95% CI, 27.2%–51.2%) compared to those using Amsel criteria (29.9%, 95% CI, 24.1%–36.5%). Diagnosing BV by presence of clue cells as only criterion (study of *Verbruggen et* *al.* using the KOPAC system included) or by modified Amsel criteria showed the lowest prevalence (13.6%, 95% CI, 5.1%–22.1%). These data are visualized in the box plots of figure 2. Prevalence of BV in studies conducted in the Netherlands was remarkably low, ranging from 3.14% in asymptomatic women to 5.48% in women with symptoms (or indication smear).

Differences in BV prevalence were also significant according to the study population (p = 0.02). Pooled BV prevalence in cervical screening studies [13], [26], [27], [40] (13.2%, 95% CI; 0–26.3%) was significantly lower compared to the other studies, including women referred to colposcopy clinic and attending obstetric/gynaecological clinics (30.0%, 95% CI; 23.8%–36.1%). As all but one screening study used only clue cells for detecting BV, the observed low prevalence for studies using clue cells only, may be well explained by this fact. BV prevalence did not differ significantly between studies carried out in developed countries (28.0%, 95% CI; 20.8%–35.3%) versus studies carried out in developing countries (23.5%, 95% CI; 9.6%–37.5%). However, these pooled prevalences cannot be extrapolated easily due to the observed heterogeneity, the differences in study design and the small number of studies from developing countries included in the analysis.

### Association between Bacterial Vaginosis and Cervical Lesions

**Figure 3:**
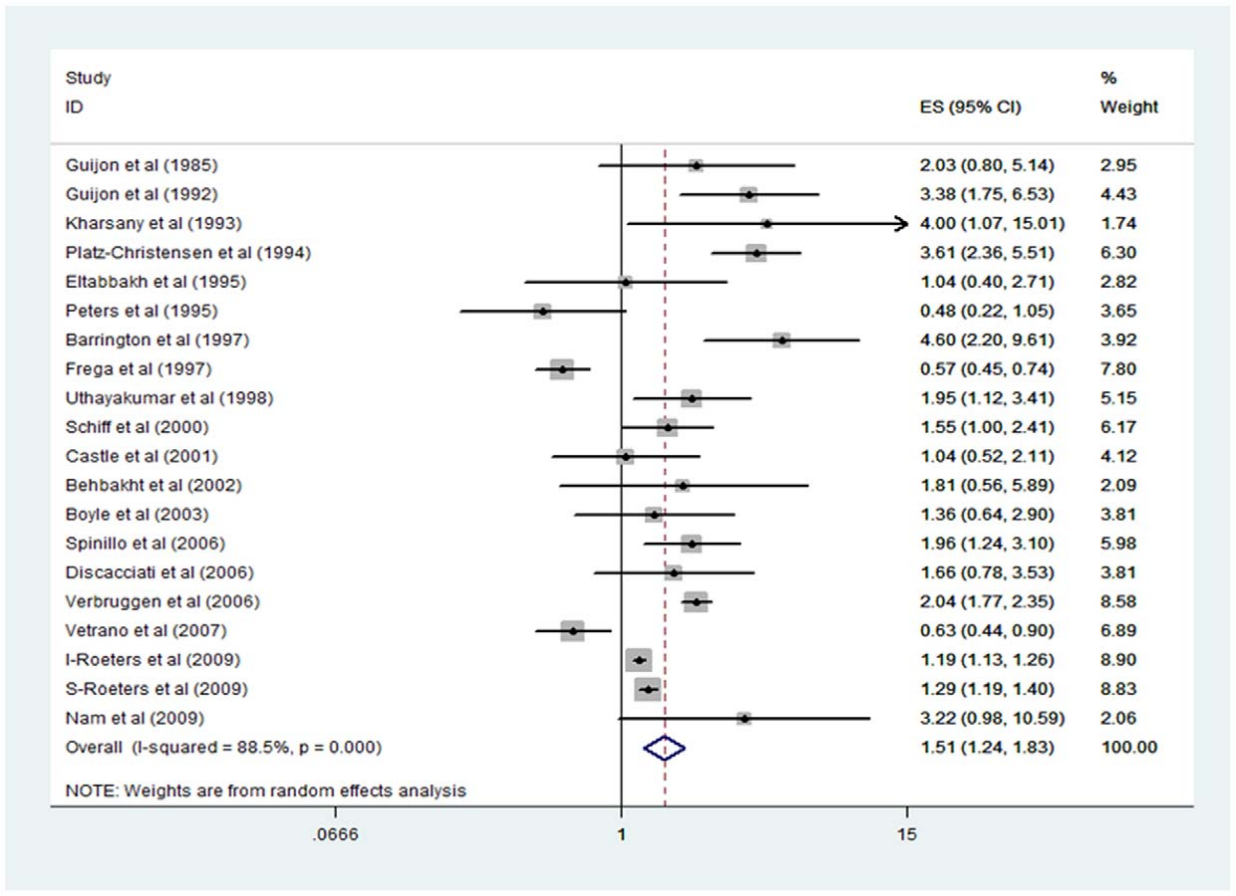
Forest plot of studies included in meta-analysis BV – CIN. Each study is represented by a black square and a horizontal line, which corresponds to the odds ratio (OR) and its symmetric 95% confidence interval (CI). The area of the square reflects the weight each study contributes to the meta-analysis. The diamond at the bottom of the graph represents the combined OR and its 95% CI, calculated using a random effects model. The solid vertical line corresponds to no association (OR 1.0), the dotted vertical line to the combined OR (1.51). The OR (or estimates ES), 95% CI and weights are also given in tabular form.

Analysis of the association between BV and pre-cancerous cervical lesions showed that CIN or SIL prevalence was significantly higher in BV positive women in 10 out of 20 different estimates compared to women without BV. Figure 3 represents the odds ratios (OR) and their 95% confidence intervals (CI) of the association between BV and CIN, the weight given to each study in a random effects model, and the summary estimate with 95% CI. ORs in different studies ranged from 0.48 [18] to 4.60 [10]. The combined OR for included prevalence studies was 1.51 (95% CI, 1.24–1.83, p<0.05), indicating a significant positive association between BV and CIN.

The studies conducted in the Netherlands using the KOPAC system [26], [27] mention a number of smears, but given the overlapping study periods, it cannot be ruled out that these studies included the same women multiple times. Without more information one cannot correct for this in a satisfactory way. Yet, meta-analysis with omission of these studies still yielded a significant positive pooled OR of 1.62 (95% CI 1.10–2.38). Hence the inclusion of these studies did not alter the conclusion of this paper significantly.

The funnel plot showed little asymmetry, and the Beggs rank correlation test did not show any significant indication for publication bias (z = 0.52, p = 0.604). This observation, together with the fact that 10 out of 20 studies reported a non-significant association, render publication bias rather unlikely. To investigate the influence of a single study on the overall meta-analysis estimate, an influence analysis was conducted. None of the studies was highly influential and the OR varied little, ranging from 1.41 (after excluding the study by Platz-Christensen et al [13]) to 1.62 (after excluding the study by Frega et al [41]).

**Figure 4:**
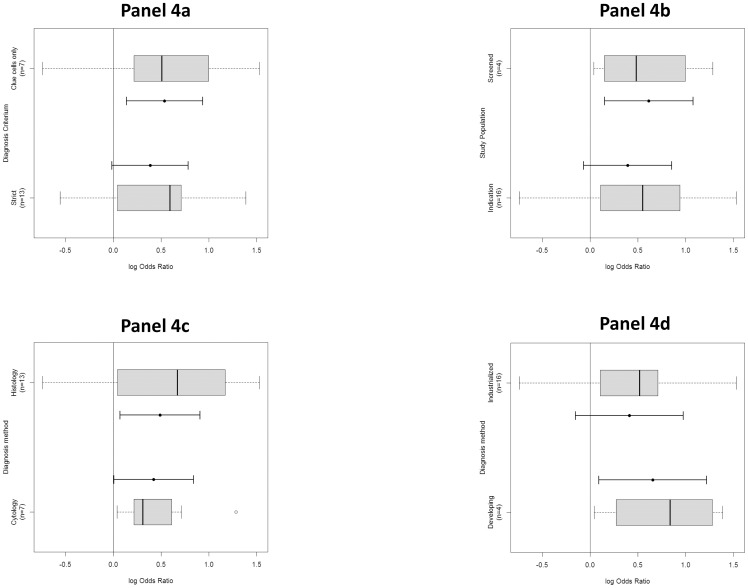
Box plots according to BV diagnostic criteria and study population. Each box plot represents a summary of 5 data: 25th and 75th percentile or inter-quartile range of the data (left and right edge of box, respectively), the median (vertical band near the middle of the box), the minimum and maximum data value (ends of horizontal lines or whiskers). Full horizontal lines represent OR and its 95% CI according to BV diagnosis (left) and stratified by study population (right), estimated by random effects regression model. *Figure 4a*: Difference in odds ratio (logarithmic scale) depending on BV diagnostic criteria: Clue cells only (one study using Modified Amsel, i.e. presence of clue cells and positive amine whiff test) versus more stringent criteria (strict), including Nugent and Amsel. *Figure 4b*: Difference in odds ratios (logarithmic scale) depending on study population, stratified as screened women and women with an indication smear (e.g. referred for colposcopy or obstetric/gynaecologic clinic attendees). *Figure 4c:* Difference in odds ratio (logarithmic scale) depending on CIN diagnostic criteria: Cytology versus Histology. *Figure 4d:* Difference in odds ration (logarithmic scale) depending on developing state of the country, stratified as developing and industrialized.

The wide range of reported odds ratios among included studies suggested heterogeneity and this was confirmed according to the Cochran's Q test (χ^2^ = 164.7, p<0.01). About 88.5% of the total variation could be explained by heterogeneity between samples (I^2^ = 88.5). To explore sources of heterogeneity and examine possible explanations for the differences in the reported associations between BV and CIN among studies, meta-regression was performed. The included studies were stratified according to BV diagnostic criteria (clue cells and modified Amsel versus stringent criteria, including Nugent and Amsel), CIN diagnostic criteria (histology versus cytology), country (developed versus developing countries) and according to the study population (screened versus indication smears, including women referred to colposcopy clinic, obstetric/gynaecological attendees or a mixed population). None of the stratification factors resulted in a significant difference in OR (Figure 4). Although heterogeneity was significant, this could not be contributed to any of the stratification factors.

## Discussion

Cervical carcinogenesis must involve the presence of additional promoting factors, since only a minority of patients harbouring HPV develop cervical dysplasia [3]. BV has been suggested as an intriguing possible co-factor in cervical carcinogenesis. Previous studies examining the relationship between BV and CIN, however, have rendered conflicting results. This meta-analysis with over 10.000 women and in addition a database of more than one million cervical smears, is to our knowledge the first study confirming a positive association between BV and cervical pre-cancerous lesions, with a significant overall estimated odds ratio of 1.51.

The role of BV as a co-factor in the natural history of HPV infection and related disease remains largely elusive. A putative explanation might be the fact that BV promotes – as noted for most sexually transmitted infections [17] – the acquisition and persistence of HPV infection. In a previous meta-analysis a positive association between BV and cervical HPV infection was confirmed (OR 1.43; 95% CI, 1.11–1.84) (Gillet et al, in press). Furthermore, BV is associated with profound changes in the physicochemical and immunological environment of the vaginal niche. It has been suggested that an elevated vaginal pH, as present in BV, may arrest squamous metaplasia in the post-pubertal cervix and prolong the period in which the transformation zone is vulnerable to agents promoting dysplasia such as HPV [46]. Da Silva et al. described an increased frequency of BV and Chlamydia trachomatis in pregnant women with HPV infection [47]. Biochemical changes in vaginal secretions of women with BV include production of metabolic by-products, such as propionate and butyrate, capable of damaging epithelial cells. In addition, the BV-associated anaerobes release volatile amines (especially putrescine, trimethylamine and cadaverine) [48], responsible for the characteristic fishy malodour [15]. Amines appear in the vaginal environment after conversion of amino acids produced by abundance of anaerobes, and form in combination with nitrites (produced by nitrate reducing bacteria) nitrosamines [49]. These carcinogenic compounds are capable of forming DNA adducts and consequently mutagenic events [50]. Previous investigations suggest that local accumulation of nitrosamines during episodes of BV may induce cell transformation of the cervical epithelium, in concert with other oncogenic agents like HPV infection [10], [38], [46], [49], [51], [52].

Alternatively, alterations in inflammatory cytokine profile present in a disturbed vaginal environment could promote development of cervical lesions [53]. In a prospective study of Tavares-Murta and colleagues, patients with BV and CIN presented a similar local cervical immune profile, as assessed by cytokine (IL-6, IL-8 and IL-10) and nitric oxide (NO) concentrations [54]. On the other hand, it has been reported that cervical inflammation (leading to genotoxic damage through oxidative metabolites) is associated with CIN, and may be a cofactor for high-grade cervical lesions in HPV-infected women [36]. Since BV frequently coexists with cervicitis [17], a disturbed vaginal microflora might therefore indirect predispose to cervical dysplasia.

Another important additional co-factor in cervical carcinogenesis could be the relative absence of hydrogen peroxide (H_2_O_2_)-producing lactobacilli. Bauer et al elaborated a hypothetical model for lactobacilli-mediated control of cancer, in which selective apoptosis induction represents the key element of the lactobacilli-mediated antitumor defense [55]. He suggested that H_2_O_2_-producing lactobacilli and peroxidase in the vagina of healthy women and the consequently generation of hypochlorous acid (HOCl), is responsible for creating a balanced microbicidal vaginal environment and represents a natural antitumor system. If transformed cells appear in the vaginal mucosa, they will be driven into selective apoptosis by interaction of the preformed HOCl with target cell-derived reactive oxygen species (superoxide anions), which leads to the site-specific generation of highly reactive hydroxyl radicals [55].

Some methodological limitations need to be considered. First of all, most included studies had a cross-sectional design, where data on prevalence of BV and cervical lesions were gathered simultaneously, rather than longitudinally. Therefore, this analysis is liable to reverse causation bias and prohibits concluding that BV plays a causal role in cervical carcinogenesis. BV may influence onset and progression to cervical pre-cancerous lesions, but it is also plausible that cervical dysplasia favours conditions for disruption of the normal vaginal environment and promotes an abundant growth of anaerobes. Since the vaginal environment is considered to be influenced by various factors, such as hormones and the state of the vaginal mucosa, gynaecological diseases may affect the growth of the vaginal microflora. Only a cohort study can determine which condition precedes the other. In this systematic review, only two incidence studies were found. In a study conducted by Lehtovirta et al [20] BV was associated with a significantly increased risk of CIN in univariate analysis (Hazard ratio (HR) 1.85, 95% CI 1.04–3.28, p = 0.04) and approached significance in multivariate analysis (HR 2.32; 95% CI 0.95–5.65). In another retrospective cohort-study of Engberts et al [25], women with dysbacteriosis were significantly more likely to have LSIL and HSIL in their follow-up smear (OR 1.89, 95% CI 1.42–2.52).

The question remains whether there is a causal relation between BV and cervical pre-cancerous lesions, or whether both conditions co-occur in sexually active women. It is known that a number of socio-demographic and lifestyle behavioural factors influence the risk of BV and cervical pre-cancerous lesions in a similar fashion. Although not considered an STI in its usual sense, BV mirrors this profile [56], and is associated with sexual activity and thus a candidate for an epidemiological association with CIN. Most studies examining the association between BV and CIN failed to take into account confounding factors, such as presence of HPV, sexual habits and cigarette smoking. Only three included studies performed multivariate analysis and adjusted for confounding factors in examining the association between BV and CIN (Castle et al [36], Schiff et al [14], Spinillo et al [21]). However, these few studies yielded conflicting results.

Although meta-regression could not clarify heterogeneity of results, a number of variables could contribute to the variety of association between BV and cervical lesions. Most prominent, BV prevalence varied according to the study population. Various social habits and ethno-geographical risk factors may explain the wide BV prevalence range observed (3 to almost 50%). It is well recognized that prevalence of BV in African women is among the highest worldwide. Therefore, it would be interesting to evaluate the association between BV and cervical lesions in African women, since we may expect a more pronounced effect. Only one study included in this meta-analysis (Kharsany et al, 1993) was conducted in South-Africa. Indeed, BV prevalence, diagnosed by Amsel criteria, was high (37.5%) and although the sample size was rather small, the estimated odds ratio was the second highest of all included studies (OR 4.0; 95% CI, 1.07–15.1) [12]. Technical biases, subjectivity, sensitivity and specificity of diagnostic methods could also contribute to detected heterogeneity. Especially for diagnosing BV, criteria varied strong among the studies. Two included studies conducted in the Netherlands used a unique coding system (KOPAC), defining BV as dysbacteriosis [26], [27]. Although dysbacteriosis is associated with the clinical syndrome BV, differences between the two entities certainly exist, since dysbacteriosis is a 100% morphological (light microscopic) diagnostic method as opposed to the clinical Amsel criteria. However, this meta-analysis was also conducted without these studies using the KOPAC system, yielding still a positive and more pronounced association (OR 1.62; 95% CI 1.10–2.38).

Further, this meta-analysis was limited to that of published studies, which could have caused publication bias, resulting from tendency to selectively publish results that are statistically significant. However, half of the included studies showed no significant association between BV and cervical pre-cancerous lesions, and Beggs rank correlation test did not give any indication of a possible publication bias either. In addition, the literature review was limited to English language studies found in two major databases, i.e. Pubmed and Web of Science.

In conclusion, this meta-analysis confirms a positive association between BV and cervical pre-cancerous lesions and emphasizes the potential role of a disturbed vaginal microflora in gynaecologic complications. BV is one of the most common conditions of child-bearing aged women worldwide, and considering a possible synergy of an imbalanced vaginal environment with cervical pre-neoplasia, it is clear that greater attention needs to be given to this condition. These results support the need for prospective cohort-studies addressing the interrelationships between BV and CIN, where sensitive and specific diagnostic methods are used, and were confounding factors, are taken into account. If BV plays a promoting role in the development of cervical cancer, then women with a history of recurrent or persistent BV should be eligible for closer follow-up, and restoring the vaginal microflora should in that case be a promising answer.

## Supporting Information

Checklist S1 **PRISMA Checklist for systematic review and meta-analysis.**
(DOC)

Table S1 **Raw data of studies included in meta-analysis bacterial vaginosis – cervical intraepithelial neoplasia.**
(DOC)
