# Supplementary material for: Association between Bacterial Vaginosis and Cervical Intraepithelial Neoplasia: Systematic Review and Meta-Analysis
Source: PLoS One. 2012 Oct 2;7(10):e45201. doi: 10.1371/journal.pone.0045201 (PMC3462776; doi:10.1371/journal.pone.0045201)
Supplement: Table S1 — Raw data of studies included in meta-analysis bacterial vaginosis – cervical intraepithelial neoplasia. (DOC) [file pone.0045201.s002.doc]

Table S1: *Raw data of studies included in meta-analysis BV – CIN*

| **Year of Publication** | **Authors** | **BV+ CIN+** | **BV+ CIN-** | **BV- CIN+** | **BV- CIN-** | **Total** |
| --- | --- | --- | --- | --- | --- | --- |
| 1985 | Guijon et al^41^ | 14 | 15 | 17 | 37 | 83 |
| 1992 | Guijon et al^11^ | 51 | 17 | 55 | 62 | 185 |
| 1993 | Kharsany et al^12^ | 14 | 4 | 14 | 16 | 48 |
| 1994 | Platz-Christensen et al^13^ | 31 | 586 | 80 | 5453 | 6,150 |
| 1995 | Eltabbakh et al^39^ | 6 | 138 | 16 | 383 | 543 |
| 1995 | Peters et al^18^ | 39 | 11 | 193 | 26 | 269 |
| 1997 | Barrington et al^10^ | 33 | 12 | 58 | 97 | 200 |
| 1997 | Frega et al^40^ | 180 | 248 | 324 | 256 | 1,008 |
| 1998 | Uthayakumar^43^ | 29 | 43 | 57 | 165 | 294 |
| 2000 | Schiff et al^14^ | 50 | 111 | 62 | 214 | 437 |
| 2001 | Castle et al^35^ | 20 | 29 | 37 | 56 | 142 |
| 2002 | Behbakht et al^36^ | 10 | 15 | 7 | 19 | 51 |
| 2003 | Boyle et al^37^ | 12 | 96 | 20 | 218 | 346 |
| 2006 | Discacciati et al^38^ | 20 | 13 | 90 | 97 | 220 |
| 2006 | Spinillo et al^21^ | 36 | 83 | 81 | 366 | 566 |
| 2007 | Vetrano et al^44^ | 91 | 119 | 161 | 133 | 504 |
| 2009 | Nam et al^42^ | 53 | 3 | 384 | 70 | 510 |
| 2009 | Roeters et al^26^ | Raw data not mentioned | | | | 1,008,879 |
| 2006 | Verbruggen et al^27^ | 212 | 14,518 | 3,061 | 427,289 | 445,080 |
